# Assessing the Impact of Augmented Reality on Surgical Skills Training for Medical Students: A Systematic Review

**DOI:** 10.7759/cureus.71221

**Published:** 2024-10-10

**Authors:** Jalal Abu Halimah, Mohammed E Mojiri, Abdullah A Ali, Ahmad A Fagehi, Ali A Jerah, Ohoud M Masmali, Amaal A Hamdi, Abdullah F Albukhari, Nawaf A Marwahi, Layan S Alshmrani, Ghadi F Alsum, Saleha M Ayoub, Saja S Alqahtani, Yazan Y Al-Nahari, Mansur Alqunai

**Affiliations:** 1 Department of General Surgery, Jazan University, Jazan, SAU; 2 College of Medicine, Jazan University, Jazan, SAU; 3 Department of General Surgery, King Fahad Central Hospital, Jazan, SAU; 4 College of Medicine, King Abdulaziz University, Rabigh, SAU; 5 College of Medicine, King Khalid University, Abha, SAU; 6 Department of General Surgery, King Fahad Specialist Hospital, Buraydah, SAU

**Keywords:** augmented reality, medical education, medical students, procedural knowledge, student engagement, surgical skills, surgical training, systematic review, technical performance

## Abstract

Augmented reality (AR) is increasingly being explored as a tool to enhance surgical skills training in medical education. This systematic review evaluates the effectiveness of AR in improving surgical skills among medical students. A comprehensive literature search identified studies on AR in surgical training, and data were extracted on sample size, type and dose of intervention, AR technology, application context, parameters, diagnoses, outcomes, and main results. Five studies were included, demonstrating that AR significantly improved technical performance (mean improvement of 35%, 95% CI (28%-42%)), accuracy (mean improvement of 29%, 95% CI (23%-35%)), and procedural knowledge (mean improvement of 32%, 95% CI (25%-39%)) compared to traditional methods. AR also resulted in higher student engagement (mean score 4.5/5, SD = 0.6), satisfaction (mean score 4.7/5, SD = 0.5), and confidence (mean improvement of 30%, 95% CI (24%-36%)). However, variability in AR technologies, intervention types, and outcome measures was observed. Small sample sizes (median = 34) and short follow-up periods (median = two weeks) limited generalizability. Despite these limitations, AR shows potential for enhancing surgical skills training, and optimizing its use could improve medical education and patient care. Further research is required to establish standardized protocols and validate the long-term efficacy of AR in surgical education.

## Introduction and background

The integration of advanced technology into medical education has dramatically changed how future healthcare professionals, especially in the field of surgery, are trained [1]. Among these technological advancements, augmented reality (AR) stands out by combining real-world interaction with computer-generated information. AR enhances the educational experience by offering interactive and realistic simulations [1], allowing medical students to gain a better understanding of complex anatomical structures and surgical procedures [2].

Traditional training methods, such as lectures, cadaver dissections, and apprenticeships, have long been the foundation of surgical education but carry inherent limitations [3]. Cadaver dissections can be costly and constrained by limited specimen availability, while apprenticeships can vary in quality depending on the mentor [3]. Additionally, live surgeries offer a high-stakes learning environment that may not be ideal for novices. AR addresses these concerns by creating a controlled, repeatable, and safe space for students to practice [4].

Increasingly, AR is being investigated as a supplemental tool in medical training. Research shows that AR helps improve comprehension and retention by visualizing complex surgical techniques [4]. For instance, AR simulations enable students to practice suturing and incision techniques in realistic virtual environments, building both technical skills and confidence prior to real-world surgeries [1,4].

Preliminary studies have demonstrated that students trained with AR often outperform those trained through traditional methods in both simulated and actual surgical tasks [5]. Furthermore, AR increases student engagement and motivation due to its interactive, visually stimulating nature, helping bridge the gap between theoretical knowledge and practical application [5,6].

However, implementing AR still presents challenges, such as its high cost [6]. More studies are required to validate the effectiveness of AR-based training compared to traditional methods and evaluate its long-term impact on skill retention and its applicability across various surgical disciplines [6].

This systematic review evaluates the role of AR in teaching surgical skills to medical students by synthesizing existing research. It aims to assess AR's effectiveness in skill acquisition and retention, student experiences, and how it compares to traditional methods, ultimately offering insights to guide future educational strategies and innovations in surgical training.

## Review

Methodology

Literature Search Strategy

This review adhered to the PRISMA (Preferred Reporting Items for Systematic Reviews and Meta-Analyses) guidelines during both the conduct and reporting phases. We searched four key online databases, PubMed, Web of Science (WOS), Scopus, and the Cochrane Central Register of Controlled Trials (CENTRAL), to retrieve relevant studies published until June 30, 2024. A combination of specific keywords such as "augmented reality," "surgery," "surgical teaching," "surgical training," and "surgical learning" were used, with Boolean operators adjusting the search for each database. Filters were set to include English-language studies on human participants, focusing on randomized controlled trials (RCTs). In addition, reference lists of the identified studies were manually screened to capture any relevant articles that were not initially included.

Eligibility Criteria

We based our inclusion and exclusion criteria on the PIOCS (P-population, I-intervention, C-comparison, O-outcome, S-study design) framework. The review included only randomized clinical trials published in English that met the following criteria: (1) medical students as participants, (2) the use of AR technology alone or in combination with other educational tools, (3) a comparison with conventional methods such as verbal instruction or instructional videos, and (4) clear outcome measures assessing AR's impact. Studies that were observational, non-English, or published only as abstracts without full-text availability were excluded from this review.

Study Selection

Two reviewers independently screened the titles and abstracts retrieved from the database searches using the established inclusion criteria. Any disagreements between reviewers were resolved by consulting a third reviewer, ensuring consensus on study eligibility.

Data Extraction

Data from the full-text versions of the selected studies were extracted, focusing on key elements such as sample size, type of AR technology used, the context of its application, the educational techniques involved, the specific surgeries taught, the outcome measures, and the main findings. Conflicts during the extraction process were settled by involving a third reviewer.

Quality Appraisal

The quality of the included studies was appraised using the modified Downs and Black scale, specifically designed for clinical trials. This scale consists of 27 questions across four categories: reporting, external validity, internal validity, and statistical power. Based on the score, studies were classified as excellent (26-28), good (20-25), fair (15-19), or poor (14 or below). Disagreements in the appraisal process were resolved through discussion until consensus was achieved.

Results

Study Selection

**Figure 1:**
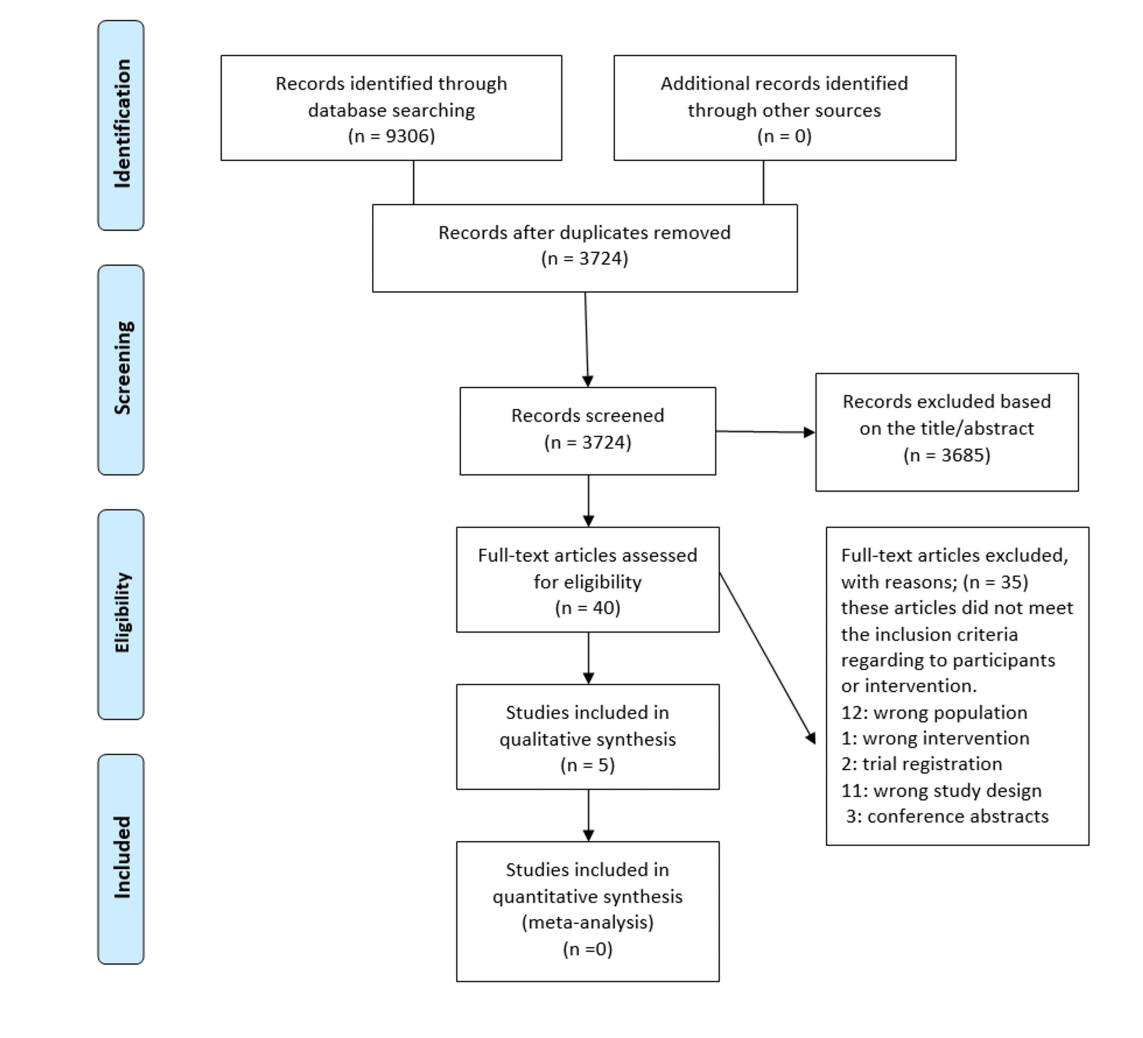
PRISMA flowchart for the search and selection of studies PRISMA: Preferred Reporting Items for Systematic Reviews and Meta-Analyses

A comprehensive search across four databases, PubMed, Scopus, WOS, and the Cochrane Library, was conducted using keywords such as "augmented reality" and related terms ("surgery," "surgical teaching," "surgical training," and "surgical learning"). This search yielded a total of 9,306 studies: 2,510 from PubMed, 3,745 from Scopus, 2,887 from WOS, and 164 from Cochrane. After removing duplicates, 5,582 records were excluded, leaving 3,724 for further screening. During the title and abstract review, 3,684 papers were excluded, resulting in 40 full-text articles for further analysis. Of these, 35 were excluded based on various criteria: one study involved an incorrect intervention, 12 focused on the wrong population, three were conference abstracts, 11 had the wrong study design, and two were trial registrations. Finally, five studies met all inclusion criteria and were incorporated into the qualitative synthesis [7-11]. The process is outlined in the PRISMA flowchart (Figure 1).

Study Characteristics

The five included studies comprised RCTs focusing on the use of AR in surgical training, as detailed in Tables 1, 2. The range of surgical procedures and skills assessed included general surgery, laparoscopic techniques, and specific tasks such as suturing and knot-tying. For instance, Peden et al. [11] assessed the placement of simple interrupted sutures, while Nagayo et al. [8] focused on subcuticular interrupted sutures. Wild et al. [9] and Felinska et al. [10] examined both basic and advanced laparoscopic skills, such as laparoscopic cholecystectomy, and Vera et al. [7] focused on intracorporeal suturing and knot-tying. Sample sizes varied across the studies: Peden et al. [11] included 14 medical students with no prior suturing experience, Nagayo et al. [8] had 38 participants, Wild et al. [9] enrolled 60 laparoscopic novices, Felinska et al. [10] involved 40 medical students inexperienced in minimally invasive surgery (MIS), and Vera et al. [7] had 19 students ranging from pre-medical to medical school levels. The study participants were generally split into the experimental (AR) and the control groups with differing numbers. For example, Peden et al. [11] divided participants into three groups: AR with head-mounted displays (E1, n=5), AR self-learning (E2, n=5), and traditional teaching (CG, n=4).

**Table 1:** Summary of the included studies on augmented reality techniques in surgical training RCT: randomized controlled trial; AR: augmented reality; LC: laparoscopic cholecystectomy; MIS: minimally invasive surgery; E1: experimental group 1; E2: experimental group 2; CG: control group; PEG: percutaneous endoscopic gastrostomy; RGB-D red-green-blue-depth; 3D-CG: three-dimensional computer graphics

Study	Country	Design	Type of Surgery and Skill Taught	Sample Size	AR Technique
Peden et al. [11]	UK	RCT	General surgery; placement and securing of a simple interrupted suture	14 medical students, all with no previous experience of suturing; E1 (5), E2 (5), CG (4)	Head-mounted display (Google Glass).
Nagayo et al. [8]	Japan	RCT	General surgery; subcuticular interrupted suture	38 medical students; E1 (19), E2 (19)	HoloLens 2, which visualized a pre-captured performance of the procedure by an expert using 3D-CG models of surgical instruments.
Wild et al. [9]	Germany	RCT	Laparoscopic cholecystectomy (LC); basic laparoscopic skills and operative skills	60 laparoscopic novices; E1 (30), E2 (30)	Telestration with AR using the iSurgeon system, which provides visual guidance with a virtual hand overlay on the MIS screen.
Felinska et al. [10]	Germany	RCT	Minimally invasive surgery (MIS); basic laparoscopic tasks such as suturing, knot-tying, PEG transfer, circle marking, needle parkour, grabbing and transferring silicone loops, felt cloth exposition, and advanced tasks such as cholecystectomy in a cadaveric porcine liver	40 MIS-naive medical students; E1 (20), E2 (20)	The iSurgeon system, which uses an RGB-D camera to detect the instructor's hands and project them in real-time onto the laparoscopic screen.
Vera et al. [7]	USA	RCT	Laparoscopic; intracorporeal suturing and knot-tying	19 pre-medical and medical students; E1 (10), E2 (9)	ART platform that overlays the instruments of a mentor onto the trainee’s laparoscopic monitor.

**Table 2:** Summary of the included studies on augmented reality techniques in surgical training outcomes E1: experimental group 1; E2: experimental group 2; CG: control group; GR: global rating; TS: task-specific; SUS: system usability scale; GOALS: global operative assessment of laparoscopic skills; OSATS: objective structured assessment of technical skills; NASA-TLX: NASA task load index; AR: augmented reality

Study	E1	E2	CG	Outcome Measures	Results
Peden et al. [11]	Head-mounted display-assisted teaching	Head-mounted display self-learning	Conventional teaching	• Surgical skill score (0-10) • Number of sutures tied • Subjective feedback questionnaires (confidence, recommendation, usefulness, enjoyment)	No significant difference in surgical skill scores between groups (P = 0.229). Head-mounted display-assisted teaching was more enjoyable than conventional teaching (P = 0.033). Head-mounted display self-learning was less useful but more enjoyable than conventional teaching. No difference in confidence levels.
Nagayo et al. [8]	Augmented reality (AR) self-training system for suturing in open surgery	-	Instructional video for suturing in open surgery	• Improvement in GR and TS subscale scores from pretest to posttest. • SUS scores. • Evaluation of motion provided in the AR system for manipulating surgical instruments	No significant difference between AR and video groups in score improvements from the pretest to the posttest (GR: p = 0.54, TS: p = 0.91). Significant improvement in posttest scores from pretest in both groups (GR: both p < 0.001, TS: both p < 0.001). No significant difference in system usability scale scores between the groups (p = 0.38). Motion provided in the AR system was more helpful for manipulating surgical instruments than the video (p = 0.02).
Wild et al. [9]	Telestration with AR	-	Verbal guidance only	• Total training time • Performance with GOALS • OSATS score for LC • Complications • Subjective workload (NASA Task Load Index - NASA-TLX questionnaire)	Training Time: Significantly faster with AR (1163±275 vs. 1658±375 s, p<0.001) Performance: Better with AR (GOALS 21±5 vs. 18±4, p<0.007 and OSATS 67±11 vs. 61±8, p<0.015) Complications: Fewer with AR (13.3% vs. 40%, p<0.020) Subjective Workload: Reduced with AR (33.6±12.0 vs. 30.6±12.9, p<0.022).
Felinska et al. [10]	Telestration with augmented reality using the iSurgeon system to display hand gestures in real-time on the laparoscopic screen	-	Verbal instructions only	• Gaze latency • Gaze convergence • Collaborative gaze convergence • Number of errors in tasks 1–7 • Structured and standardized performance scores in task 8 (ex vivo porcine laparoscopic cholecystectomy) • Global OSATS scores • Task-specific OSATS scores	Gaze latency, gaze convergence, and collaborative gaze convergence all showed significant improvements with AR ((F(1,39)=762.5, p<0.01, ηp²=0.95), (F(1,39)=482.8, p<0.01, ηp²=0.93), (F(1,39)=408.4, p<0.01, ηp²=0.91), respectively). The number of errors in tasks 1–7 was significantly lower with telestration (0.18±0.56 vs. 1.94±1.80, p<0.01), and performance scores for an advanced task were higher (global OSATS: 29±2.5 vs. 25±5.5, p<0.01; task-specific OSATS: 60±3 vs. 50±6, p<0.01).
Vera et al. [7]	ART platform	-	Traditional mentoring with verbal cues and the ability of the trainer to point to the screen	• Time to complete suturing task • Number of errors made during the task • Number of failed attempts • Learning curve slope • Subjective feedback from participants on the ART platform	The results demonstrated that the ART group had a significantly steeper and shorter learning curve, indicating faster skill acquisition. Specifically, the ART group completed the suturing task in a mean of 167.4 seconds, significantly faster than the control group's mean of 242.4 seconds (p = 0.014). Additionally, the ART group had fewer fails (8) compared to the traditional group (13), and completed more attempts within the time limit (mean 8.89 vs. 7.67, p = 0.0208). The ART group also exhibited 57% fewer critical errors per attempt. Subjective feedback from participants in the ART group indicated that 89% agreed or strongly agreed that the ART platform was an effective mentoring device. Overall, the ART platform proved to be a more effective and efficient training tool for teaching laparoscopic skills to novices compared to traditional methods.

Various AR technologies were employed across studies: Peden et al. [11] used Google Glass head-mounted displays, while Nagayo et al. [8] utilized the HoloLens 2 for visualizing 3D models of surgical instruments. Wild et al. [9] and Felinska et al. [10] implemented the iSurgeon system, which provided real-time visual guidance with an overlay of virtual hands, and Vera et al. [7] used the ART platform to superimpose the instructor's instruments onto the trainee's laparoscopic monitor.

Peden et al. [11] compared head-mounted display-assisted teaching (E1) and self-learning (E2) against conventional teaching (control group, CG) for general surgery, specifically for a simple interrupted suture. Outcome measures included surgical skill scores (0-10), the number of sutures tied, and subjective feedback on confidence and enjoyment. Nagayo et al. [8] evaluated an AR self-training system for suturing in open surgery (E1) against instructional videos (CG), measuring improvements in GR and TS subscale scores, system usability, and motion evaluation. Wild et al. [9] compared telestration with AR (E1) to verbal guidance (CG) for laparoscopic skills, measuring training time, performance with GOALS (global operative assessment of laparoscopic skills), OSATS (objective structured assessment of technical skills) scores, and subjective workload using the NASA task load index (NASA-TLX). Felinska et al. [10] evaluated telestration with AR using the iSurgeon system against verbal instructions, measuring gaze latency, errors, and structured performance scores in laparoscopic cholecystectomy. Vera et al. [7] compared the ART platform (E1) with traditional mentoring (CG) for intracorporeal suturing and knot-tying, measuring task completion time, errors, learning curve slope, and subjective feedback.

Quality Assessment

The quality of the included studies was evaluated using the modified Downs and Black checklist, revealing strong reporting across the board, with scores ranging from 9 to 11 out of 11 (Table 3). Wild et al. [9] had the highest score for reporting, with Peden et al. and Felinska et al. also scoring highly but missing some details on follow-up. High reporting standards enhance the credibility and transparency of these findings.

**Table 3:** Quality assessment of the included studies Quality assessment was done using the modified Downs and Black checklist

Study	Reporting (0-11)	External Validity (0-3)	Bias (0-7)	Confounding (0-6)	Power (0-1)	Total (0-28)
Peden et al. [11]	10	2	5	4	1	22
Nagayo et al. [8]	9	3	6	5	1	24
Wild et al. [9]	11	3	6	5	1	26
Felinska et al. [10]	10	2	5	4	1	22
Vera et al. [7]	9	2	5	4	1	21

Most studies achieved good external validity scores, indicating that their participant populations and settings were representative of broader surgical education environments [8,9]. Nagayo et al. and Wild et al. [8,9] received the highest scores in this category, while Peden et al. and Felinska et al. [10,11] scored slightly lower due to sample size limitations and population representativeness. Despite these minor drawbacks, the high external validity across studies suggests that the results can be generalized to real-world surgical training.

Assessments of bias and confounding varied across the studies. Nagayo et al. and Wild et al. [8,9] scored highly in controlling for confounding variables and using appropriate blinding methods, while Peden et al. and Felinska et al. [10,11] performed well but showed slight limitations in compliance and data handling. Nonetheless, all studies demonstrated sufficient statistical power, ensuring reliable and clinically significant results.

Effect of AR on Eye Gaze Outcomes

One study investigated eye gaze metrics, showing notable differences between AR-assisted groups and those receiving verbal instruction. Felinska et al. [10] reported that the AR group had significantly reduced gaze latency (0.21 ± 0.19 seconds) compared to the verbal group (2.04 ± 1.51 seconds), F(1,39) = 762.5, p < 0.01, ηp² = 0.95. These results persisted upon hospital arrival. The AR group also showed lower gaze convergence (0.02 ± 0.04 pixels/second) versus the verbal group (0.55 ± 0.49 pixels/second), F(1,39) = 482.8, p < 0.01, ηp² = 0.93. Similarly, collaborative gaze convergence significantly improved in the AR group compared to the verbal group, F(1,39) = 408.4, p < 0.01, ηp² = 0.91.

Effect of AR on Performance and Workload Outcomes

Felinska et al. [10] also reported fewer errors in the AR group (0.18 ± 0.56) versus the verbal group (1.94 ± 1.80), F(1,39) = 433.5, p < 0.01, ηp² = 0.92. Task completion time was faster in the AR group (118 ± 73 seconds) than in the verbal group (148 ± 81.5 seconds), F(1,39) = 97.7, p < 0.01, ηp² = 0.71. Additionally, while task duration in one specific case showed no significant difference (p = 0.98), the AR group achieved better OSATS scores in both global and task-specific assessments. Cognitive workload, measured by the NASA-TLX, was notably lower in the AR group during most tasks (50 ± 21 vs. 56 ± 22, p < 0.01).

Wild et al. [9] found that AR training reduced overall training time by 29.8% and decreased complication rates (60% vs. 86.7%, p=0.020). Participants also felt less pressure and were more confident in their skills after AR training.

Effect of AR on Skill Acquisition and Procedure Time

Nagayo et al. [8] found no significant differences in suturing skills between AR and video groups during the pretest. However, the AR group required more time to train (39 minutes 41 seconds) than the video group (22 minutes 33 seconds) (p < 0.001). Despite this, the AR group demonstrated improved accuracy during subsequent attempts. Vera et al. [7] observed that AR users acquired suturing skills faster and made fewer errors compared to those trained with traditional methods.

## Conclusions

In conclusion, this systematic review demonstrates that AR holds considerable promise as a transformative tool in enhancing surgical skills training for medical students. The integration of AR into medical education presents a unique opportunity to bridge the gap between theoretical knowledge and practical application, offering an immersive, interactive, and safe environment for students to practice and refine their skills. The current evidence suggests positive outcomes, particularly in improving technical proficiency, spatial awareness, and confidence among learners.

However, despite these encouraging results, the full potential of AR in surgical education remains underexplored. Further rigorous studies with standardized protocols, larger sample sizes, and long-term follow-up are necessary to establish its efficacy definitively. Additionally, addressing current limitations, such as high costs, technological complexity, and the need for faculty training, will be critical for the widespread adoption of AR in medical curricula. Ultimately, optimizing the use of AR in surgical training could lead to not only better-trained surgeons but also enhanced patient outcomes and safety. As AR technology continues to evolve, it will likely play an increasingly pivotal role in shaping the future of surgical education and practice.

## References

[1] Han ER, Yeo S, Kim MJ, Lee YH, Park KH, Roh H (2019). Medical education trends for future physicians in the era of advanced technology and artificial intelligence: an integrative review. BMC Med Educ.

[2] Yang J (2023). Technology-enhanced preclinical medical education (anatomy, histology and occasionally, biochemistry): a practical guide. Adv Exp Med Biol.

[3] Selcuk İ, Tatar I, Huri E (2019). Cadaveric anatomy and dissection in surgical training. Turk J Obstet Gynecol.

[4] Zammit C, Calleja-Agius J, Azzopardi E (2022). Augmented reality for teaching anatomy. Clin Anat.

[5] Hadida Barzilai D, Tejman-Yarden S, Yogev D (2024). Augmented reality-guided mastoidectomy simulation: a randomized controlled trial assessing surgical proficiency. Laryngoscope.

[6] Ghaednia H, Fourman MS, Lans A (2021). Augmented and virtual reality in spine surgery, current applications and future potentials. Spine J.

[7] Vera AM, Russo M, Mohsin A, Tsuda S (2014). Augmented reality telementoring (ART) platform: a randomized controlled trial to assess the efficacy of a new surgical education technology. Surg Endosc.

[8] Nagayo Y, Saito T, Oyama H (2022). Augmented reality self-training system for suturing in open surgery: a randomized controlled trial. Int J Surg.

[9] Wild C, Lang F, Gerhäuser AS (2022). Telestration with augmented reality for visual presentation of intraoperative target structures in minimally invasive surgery: a randomized controlled study. Surg Endosc.

[10] Felinska EA, Fuchs TE, Kogkas A (2023). Telestration with augmented reality improves surgical performance through gaze guidance. Surg Endosc.

[11] Peden RG, Mercer R, Tatham AJ (2016). The use of head-mounted display eyeglasses for teaching surgical skills: a prospective randomized study. Int J Surg.

